# Men’s anxiety, why it matters, and what is needed to limit its risk for male suicide

**DOI:** 10.1007/s44202-022-00035-5

**Published:** 2022-03-04

**Authors:** Krista Fisher, Zac E. Seidler, Kylie King, John L. Oliffe, Steve Robertson, Simon M. Rice

**Affiliations:** 1 Orygen, Parkville, VIC Australia; 2 Centre for Youth Mental Health, The University of Melbourne, Melbourne, VIC Australia; 3 Turner Institute for Brain and Mental Health, Monash University, Victoria, Australia; 4 University of British Columbia, Vancouver, BC Canada; 5 Melbourne School of Health Sciences, The University of Melbourne, Melbourne, VIC Australia; 6 Centre for Men’s Health, Faulty of Health and Social Sciences, Leeds Beckett University, Leeds, UK; 7 Division of Nursing & Midwifery, University of Sheffield, Sheffield, UK

## Abstract

Anxiety disorders are the most prevalent mental health disorder experienced by men. If left untreated, anxiety is predictive of psychiatric disorders including depression and associated suicide risk. Despite the prevalence and impact of men’s anxiety, it remains largely overlooked in the field of men’s mental health. Globally, men are reported to have lower rates of anxiety disorders compared to women; however, these sex-differences do not reflect the complexity and nuance of men’s experiences. There is early evidence to suggest a male-type anxiety phenotype which may go undetected with generic diagnostic classifications. Masculine norms (i.e., stoicism, toughness, invulnerability) appear to be central to men’s experiences and expressions of anxiety as well as men’s help-seeking and coping behaviours. This is particularly concerning given anxiety increases men’s risk of physical and psychological comorbidities and suicide risk. The effective assessment, detection and treatment of men’s anxiety is therefore critical to improve mental health outcomes across the male lifespan. We propose three key recommendations for the field of men’s anxiety: (i) to develop a theoretical model surrounding men’s experiences of anxiety, (ii) broaden mental health resources, interventions and suicide prevention strategies to encompass men’s gendered experiences of anxiety (e.g., sentiments of shame, physical symptom manifestation), and (iii) utilise informal supports (i.e., friends and family) as an avenue of intervention to improve men’s anxiety outcomes. Without a substantial research agenda in men’s anxiety, we will fail to recognise and respond to men’s gendered experiences of anxiety and ultimately fail to reduce male suicides.

## Introduction

Despite being the most prevalent mental health disorder experienced by men, anxiety has been overlooked in mental health scholarship [1, 2]. Anxiety is often the first mental health disorder young men experience, and if left untreated it is predictive of comorbid psychiatric disorders including depression, bipolar disorder and psychosis [3]. In Australia, it is estimated 16% of females have had a diagnosed anxiety disorder in the past 12-months, in comparison to 11% of males [4]. Similar twelve-month anxiety prevalence rates are seen in other Western countries including the United States (23% of females vs. 14% of males; [5]), Canada (21% of females vs. 15% of males; [6]) and the United Kingdom [30% of females vs. 15% of males (aged 18–24 years); [7]]. Worringly in the COVID-19 context, the rates of anxiety disorders, particularly in young people, are increasing [8, 9]. Epidemiological findings are largely confined to sex differences, juxtaposing *all males* with *all females*, to imply anxiety disorders may be less prevalent and by extension less debilitating with respect to men’s overall wellbeing and social and emotional functioning [10–12]. Such sex difference analyses limit, and at times distract from thoughtful consideration of men’s diverse lived experiences of anxiety [11, 13]. In this commentary, we summarise the critical issues surrounding men’s anxiety and highlight largely unexplored implications, including associations with men’s suicidality [14, 15]. Considering early identification and the treatment of men’s anxiety is critical to improving men’s mental health outcomes across the lifespan, this commentary recommends the following agenda for future research in this developing field of inquiry. Firstly, well-established scholarship in the field of men’s depression should be leveraged to similarly develop a theoretical model surrounding men’s experiences of anxiety. Secondly, it is crucial current mental health resources, interventions and suicide prevention strategies be broadened to encompass men’s gendered experiences of anxiety. Lastly, informal supports (i.e., friends and family) should be utilised as a key avenue of intervention to improve men’s anxiety outcomes.

## Moving beyond sex-differences and prevalence rates

The true breadth and gravity of men’s anxiety is unknown, which is particularly concerning given undetected and untreated anxiety predicts future deleterious mental health outcomes [3]. Indeed, there is emerging evidence suggesting a distinct male-type anxiety phenotype [13] characterised by physical symptoms (i.e., body pains, panic attacks and headaches), chronic recurrence of symptoms, and enduring out-of-control anxious sensations [16–18]. By comparison, restlessness, being easily fatigued, difficulty concentrating, irritability, muscle tension and sleep disturbance are the current diagnostic criteria for a diagnosis of Generalised Anxiety Disorder (GAD) in the *Diagnostic and Statistical Manual of Mental Disorders Fifth Edition* (DSM-5; [19]). The putative male-type anxiety phenotype constitutes a gendered experience of anxiety that may be unique to men. Females, in comparison to males, are more likely to experience social disruption, teariness and interpersonal distress [16, 17]. These differences between male and female anxiety experiences have been largely attributed to biological factors (e.g., genetic disposition, sex specific trauma responses and increased physiological reactivity in females), psychological temperament (e.g., increased anxiety sensitivity in females) and cognitive mechanisms (e.g., lower levels of rumination and worry in males) [11, 12]. However, environmental influences such as gender socialisation, condition males and females to behave in certain gendered ways, moderating the sex-differences seen across biological, psychological and coping-mechanisms.

Traditional masculine norms prizing toughness, stoicism, self-reliance and emotional restrictiveness may be central in suppressing men’s expression of anxiety in ways that align with the DSM-5 criteria [13, 20]. More specifically, mental health professionals and men themselves are unlikely to identify anxiety if it varies from current diagnostic classifications and common understandings [21]. Masculinity has been implicated in the experience and expression of depression, as well as in men’s access and engagement with mental health care [22–24]. The role of masculine norms has predominately been considered in the context of men’s depression, with related symptoms often framed as a perceived failure to embody stoic, self-reliant masculine standards (e.g., [25]). However, similar gendered contradictions and conflicts are also likely associated with men’s anxiety.

The discord between masculine expectations and men’s anxiety is both subjective and socially conditioned [21, 26]. Men experiencing anxiety often rely on problem-solving strategies (i.e., confronting problems, searching for solutions, seeking information; [27, 28]), and attempt to deal with anxiety independently rather than utilising formal mental health services to help remedy their problems [29]. This may in part be to avoid or reduce feelings of shame, self-blame and powerlessness commonly arising from anxiety [1]. For men, shame in the context of anxiety centres on concerns around self and social acceptability leading to concealment of characteristics perceived to fall short or contradict gendered expectations of masculinity (i.e., decisiveness, rationality, coolness, calm and detachment; [30, 31]).

Anxiety may be concealed if men do not perceive it as sufficiently serious to warrant intervention. This threshold of severity, the point at which seeking help might be deemed appropriate, originates from childhood and the development of masculine identities whereby boys and young men are conditioned to embody fearlessness [30–32]. Observational studies show that boys, more so than girls, are disproportionally encouraged by parents to confront stressful and fearful stimuli [33]. This embodies a socialised rhetoric among men to ‘*face your fears*,’ positioning fear as a deviation from masculine expectations. As such, men have been traditionally socialised in Western cultures to perceive core features of anxiety, such as fear and worry, as an unwanted and unacceptable experience [10, 33]. Fearlessness is, thus, adopted as an antidote, and men’s fear is coped with in ways that do not compromise one’s masculine status (i.e., internalised, avoided or counteracted; [30, 32]). This can often compound fear and anxiety for men whereby both the trigger for anxiety (e.g., socialising with others) and the presence of fear and worry itself become overwhelming, and manifest as something to be avoided, denied and often, dampened through self-medication with alcohol or drugs.

### Men, anxiety and masculinities

This internal conflict between fear and fearlessness has been contextualised through the lens of a singular, traditional expression of masculinity. This sits as one of plural masculinities, the sum and parts of which are hierarchical in nature [34]. Multiple masculinities recognise the multifarious ways men action and embody gender in intersubjective encounters in ways which constantly shift across social situations and populations [34]. Adopting this concept of plural masculinities embraces diversity and inclusion in representations of how men express and manage their anxiety. It also allows for the naming and nuance of masculine norms that can emerge as simultaneously protective against anxiety in some contexts, and harmful in others [35, 36]. The masculine norm of toughness is an example, serving as a protective factor against anxiety by norming resiliencies and grit to counter and contest negative cognitive distortions towards oneself and the world [13, 36]. However, toughness can also encourage the concealment or masking of anxiety symptoms, ultimately delaying men’s attempts to seek help or cope with their anxiety [37, 38].

### Suicide and the implications of men’s anxiety

Considering traditional masculine norms can norm the concealment of anxiety, men experiencing these symptoms may also be at increased risk of dire outcomes including suicide. Anxiety disorders have been identified as a unique risk factor for suicidality, even when controlling for other comorbidities including depressive symptoms, major depressive disorder and substance use disorder [14, 15, 39]. Surprisingly, despite the prevalence of anxiety disorders in men, little consideration has been given to the role of anxiety in male suicide [18]. Suicide is leading cause of death amongst males aged 15–44 years in Australia, and the fourth leading cause of death amongst males aged 15–19 years worldwide [40, 41]. This is despite men’s suicide prevention becoming a global public health and economic priority [25, 42]. Men’s suicidality has long been a ‘*black box*’ in the field of men’s mental health wherein a complex range of social and psychological factors can interconnect synergistically to intensify men’s suicide risk [43].

Understanding and addressing men’s anxiety might provide one solution in preventing male suicide. Over a five-to-ten-year period, nervousness, unease and anxiety are more strongly associated with suicide attempts for men than for women [44, 45]. This may be exacerbated by specific causes and consequences of men’s anxiety experiences which include relationship breakdown [46, 47], self-harm [48], interpersonal violence and aggression [49, 50]. Given anxiety typically presents in childhood or early adolescents, the onset of suicidal ideation may therefore begin to manifest as anxiety and panic-type symptoms in some young men. As such, early identification, understanding and treatment of men’s anxiety has great scope to simultaneously intervene in, and prevent, men’s suicide outcomes.

## A way forward: developing a theoretical model for men’s anxiety

To reduce the risk of deleterious mental health outcomes associated with men’s anxiety, the gendered elements of men’s experiences, including masculinities, must be adequately considered and harnessed. The field of men’s anxiety should leverage existing scholarship (i.e., men’s depression research) to advance theoretical models and provide empirical weight to account for men’s experiences of anxiety [2, 51, 52]. Theoretical models of men’s anxiety must detail how men describe their anxiety experiences, including their patterns for expressing and managing their anxious states. They must also consider how masculine norms interconnect with men’s experiences, and how this influences and informs men's ability to cope and seek help. Incorporating cultural aspects of men’s anxiety into these theoretical models will ensure ample consideration is given to how men’s experiences and expressions of anxiety, intersect with their race, ethnicity and social determinants of health including socio-economic status [53]. Men from culturally diverse backgrounds are likely to have varied expressions and experiences of anxiety which has significant implications on the detection, management and treatment of anxiety [54]. To develop these theoretical frameworks, qualitative research must be undertaken with culturally and socially diverse populations of men. This will help contextualise the intersections of masculinities and culture across a vast array of social determinants of health to distil practices, relations and structural influences and address men’s health inequalities [55]. Such diversity and inclusion will help transcend binary sex-based research, and the ever-present reliance on overly homogenised samples of white, college-aged, middle-class men [56]. These findings will encourage increased awareness of the nuanced ways men express and manage anxiety and improve the assessment, detection, diagnosis and treatment of men’s anxiety to ensure it is gender sensitive and culturally appropriate.

### Targeting anxiety to improve men’s mental health outcomes

An increased awareness and understanding of men’s anxiety has the potential to reduce associated deleterious mental health outcomes and early mortality [44, 57]. The field of men’s anxiety needs to consider firstly, how diverse risk factors (e.g., social isolation, relationship breakdown, unemployment, substance use; [42, 58]) interconnect to intensify men’s risk of suicide, and secondly determine how these risk factors vary across differing, at-risk male sub-groups (i.e., Indigenous, LGBTQIA+ and adolescent men). Robust longitudinal research is desperately needed over short cross-sectional accounts to determine the course of anxiety across the lifespan for men. Longitudinal research will firstly, ascertain the association of anxiety with the aetiology of other co-morbid mental health issues such as depression, substance misuse, body dysmorphia and suicidality and secondly, evaluate any semblance of intervention or influence that may ultimately reduce men’s anxiety. Large cohort samples and publicly available datasets can be used to identify and track the trajectory of anxiety-related behaviours and outcomes on men’s mental health [such as the Canadian Community Health Survey (CCHS); [59], and Ten to Men: The Australian Longitudinal Study on Male Health; [60], the US National Epidemiologic Survey on Alcohol and Related Conditions (NESARC); [61]]. In addition, anxiety should be operationalised as a layered precipitating and predisposing factor for suicide. It is therefore essential that future research determines the ways in which anxiety exists as both a *cause*, and *consequence of* the above-mentioned risk factors for male suicide. Rather than depicting anxiety as a dichotomous variable (i.e., the presence or absence of anxiety), researchers should consider anxiety on a spectrum (as both an emotional experience and trait disorder) to better explore its role and interactions with numerous physical and psychological comorbidities [62].

### Broadening resources and public health promotion to encompass men’s anxiety

The absence of a gendered understanding of men’s anxiety has created a blindside in developing tailored and potentially life-saving public health campaigns. The focus has been on men’s depression, stress and resilience, which is likely due to the strong empirical links between depression and suicide and established evidence surrounding the severity and seriousness of depression [25, 63]. Existing mental health promotion resources for men’s depression (e.g., ‘HeadsUpGuys’; [64], 'Real Men Real Depression'; [65]) and interventions (e.g., 'Men's Shed'; [66], 'MATES in Construction'; [67], 'Men's Stress Workshop'; [68], Healthy@Work; [69]) should be widened to include men’s anxiety. These interventions should challenge traditional and restrictive masculine norms that have fragilized the experience of anxiety for men, or associate anxiety with femininity [70]. Interventions that attempt to normalise anxiety within a masculine context will help to destigmatise the legitimacy of male anxiety [29]. Public health campaigns targeted towards young men may be particularly effective. Young men are more likely to conform to traditional masculine norms than older cohorts [71], and in both experiencing and disclosing anxiety symptoms, perceive a cost to their masculinity, in turn expressing feelings of self-blame, failure and powerlessness [18, 29].

### Informal support: a promising intervention for men’s anxiety

Mental health resources and interventions targeting men’s informal supports (i.e., partners, family and friends) are also likely to be effective in the identification, management, treatment and ultimate prevention of men’s anxiety. Men have indicated a preference for informal support over formal mental health services to manage their anxiety [29], and men’s intimate relationships and friendships have been shown to be a space where men can talk about anxiety [2]. Informal sources of support play a significant role in improving the knowledge surrounding mental health symptoms, and positively influencing attitudes towards formal mental health care [72, 73]. The gendered frames of male help-giving influence men’s normative help-seeking attitudes and behaviours, as well as their reception towards assistance from others [74]. Challenging overtly restrictive practices of male help-giving will promote reciprocal mental health support amongst men that is devoid of indebtedness and promotes openness towards both formal and informal help-seeking [75].

Men’s anxiety interventions and resources should integrate the following principles of informal support, targeted towards men’s friends and family. Firstly, knowledge and literacy surrounding the vast diversity in men’s anxiety symptoms and disorders must be prioritised. This is particularly important given emerging evidence to suggest a male-type anxiety phenotype, which falls outside of current diagnostic classifications and mental health services [13]. Secondly, strategies that promote positive and productive help-giving behaviours must be outlined to upskill men’s friends and family in providing informal support that is both *needed* and *wanted* by men with anxiety [2]. This can only be determined by first understanding the *needs* and *wants* of men themselves and providing them opportunities to openly discuss their subjective experiences. One example of an informal support intervention could be upskilling fathers to become more effective help-givers to better detect, acknowledge and support son’s experiencing anxiety. A similar project has already been undertaken with fathers and daughters aiming to optimise girl’s physical health and social-emotional wellbeing (Dad’s And Daughters Exercising and Empowered [DADEE]; [76, 77]). Lastly, rather than replacing the need for formal support, informal support is most effective when it “proceeds, co-occurs and survives” therapeutic treatment [75]. Informal support should therefore parallel all facets of therapeutic treatment for men with anxiety, including initial help-seeking, engagement, mental health outcomes and recovery [2, 72, 75].

## Conclusion

Anxiety has a significant impact on men’s physical and psychological wellbeing [57, 78]. Prioritising early detection and intervention approaches for men’s anxiety can be improved by a gendered understanding and will benefit men’s mental health outcomes, including reduced suicide. Gender-sensitive approaches for men’s anxiety need to be co-designed, implemented and evaluated with men themselves. Interventions should attempt to de-stigmatise and norm anxiety symptoms for men (and psychological distress more broadly) privileging context and lay language to inform tools for improving the detection and management of symptoms. Treating men’s anxiety has great potential to improve men’s mental health outcomes across the lifespan and to promote positive physical, emotional, and social functioning.

## Data Availability

We did not analyse or generate any data as this manuscript is a perspective article with a theoretical approach.
